# Clinical Significance of CYFRA21-1, AFP, CA-153, CEA, and CA-199 in the Diagnosis of Lung Cancer Ocular Metastasis in Hypertension Population

**DOI:** 10.3389/fcvm.2021.670594

**Published:** 2021-09-14

**Authors:** Jing Tang, Qian-Min Ge, Rong Huang, Hui-Ye Shu, Ting Su, Jie-Li Wu, Yi-Cong Pan, Rong-Bin Liang, Li-Juan Zhang, Yi Shao, Yao Yu

**Affiliations:** ^1^Department of Endocrinology and Ophthalmology, Jiangxi Center of National Ocular Disease Clinical Research Center, The First Affiliated Hospital of Nanchang University, Nanchang, China; ^2^Department of Oncology, The Affiliated Zhuzhou Hospital Xiangya Medical College, Central South University, Zhuzhou, China; ^3^Fujian Provincial Key Laboratory of Ophthalmology and Visual Science, Medical College, Eye Institute of Xiamen University, Xiamen, China; ^4^Department of Ophthalmology, Massachusetts Eye and Ear, Harvard Medical School, Boston, MA, United States

**Keywords:** tumor markers, ocular metastasis, lung cancer, hypertension, diagnosis

## Abstract

**Purpose:** To detect lung metastases, we conducted a retrospective study to improve patient prognosis.

**Methods:** Hypertension patients with ocular metastases (OM group; *n* = 58) and without metastases (NM group; *n* = 1,217) were selected from individuals with lung cancer admitted to our hospital from April 2005 to October 2019. The clinical characteristics were compared by Student's *t*-test and chi-square test. Independent risk factors were identified by binary logistic regression, and their diagnostic value evaluated by receiver operating characteristic curve analysis.

**Results:** Age and sex did not differ significantly between OM and NM groups; There were significant differences in pathological type and treatment. Adenocarcinoma was the main pathological type in the OM group (67.24%), while squamous cell carcinoma was the largest proportion (46.43%) in the NM group, followed by adenocarcinoma (34.10%). The OM group were treated with chemotherapy (55.17%), while the NM group received both chemotherapy (39.93%) and surgical treatment (37.06%). Significant differences were detected in the concentrations of cancer antigen (CA)−125, CA-199, CA-153, alpha fetoprotein (AFP), carcinoembryonic antigen (CEA), cytokeratin fraction 21-1 (CYFRA21-1), total prostate-specific antigen, alkaline phosphatase, and hemoglobin (Student's *t*-test). Binary logistic regression analysis indicated that CA-199, CA-153, AFP, CEA, and CYRFA21-1 were independent risk factors for lung cancer metastasis. AFP (98.3%) and CEA (89.3%) exhibited the highest sensitivity and specificity, respectively, while CYRFA21-1 had the highest area under the ROC curve value (0.875), with sensitivity and specificity values of 77.6 and 87.0%, respectively. Hence, CYFRA21-1 had the best diagnostic value.

## Introduction

Among neoplastic diseases, lung cancer is the leading global cause of death in males and, for women in developed countries, mortality rates from lung cancer have now overtaken those from breast cancer (1). In recent years, the incidence of lung cancer has risen steeply in hypertension population, especially in people aged 50–70 (2). Previous experimental studies have shown that the efficacy of patients with non-small cell lung cancer during drug treatment is closely related to the risk of hypertension, confirming that early monitoring and management of hypertension may be an important step in the treatment of lung cancer (3). According to histological type, lung cancer can be classified as non-small cell lung cancer (NSCLC) (approximately 85%) and small cell lung cancer (SCLC) (approximately 15%) (4). NSCLC can be further subdivided into adenocarcinoma, squamous cell carcinoma, large cell carcinoma, and non-specific tumor type (5). Surgery is the preferred option to treat NSCLC (6); however, SCLC is conventionally treated using radiotherapy and chemotherapy (7). Most patients diagnosed with lung cancer have advanced stage disease, which contributes to their extremely poor prognosis, with the 5-year survival rates <20% (8). Lung cancer prognosis is closely related to metastasis. Common lung cancer metastasis sites are bone and brain (9), while other metastatic sites include liver (10), duodenum and pancreas (11), lymph node (12), adrenal gland (13), throat (14), and eye (15), with the eye a rare site for lung cancer metastasis. Clinical manifestations of ocular metastases include decreased vision, visual field defects, pain, muscae volitantes, and elevated intraocular pressure, among others (16); however, the condition can also be asymptomatic (17). Ocular metastases are often overlooked in early diagnosis, resulting in decreased quality of life and poor prognosis. Therefore, we conducted a retrospective study, with the aim of improving early diagnosis rates and prognosis of ocular metastasis in patients with lung cancer.

## Materials and Methods

### Ethics Statement

The Ethics Committee of the First Affiliated Hospital of Nanchang University approved this study. Subjects were fully informed before inclusion in the trial and provided informed consent. This trial was designed in accordance with the Treaty of Helsinki.

### Study Design

Lung cancer was diagnosed by histological examination of biological samples obtained by surgery or needle biopsy techniques. Hematoxylin-eosin (HE) and immunohistochemistry (IHC) staining were selected (The specific expression of thyroid transcription factor (TTF) −1 and creatine kinase (CK) −7 in lung cancer tissues indicated that the tumor tissues originated in the lung). The presence of metastases was confirmed by computed tomography (CT) and magnetic resonance imaging (MRI), and the imaging diagnosis of ocular metastases included indocyanine green angiography (ICGA), fundus fluorescein angiography (FFA), fundus photography and ocular B-ultrasound.

A total of 1,275 in lung cancer with hypertension patients admitted to our hospital from April 2005 to October 2019 were selected to participate in the trial. Among them, 58 patients with eye metastasis were the experimental (OM) group and 1,217 patients without metastasis were the control (NM) group. Inclusion criteria for the OM group were: 1) hypertension and uncontrolled blood pressure (systolic blood pressure [SBP] ≥140 mm Hg or diastolic blood pressure [DBP] ≥90 mm Hg), 2) lung cancer with ocular metastases, 3) exclusion of primary malignant tumors of the eye, 4) exclusion of benign tumors of the eye, and 4) exclusion of non-neoplastic lesions of the eye.

### Data Collection

Clinical data were retrospectively collected at the time of initial diagnosis for subsequent statistical analysis, including general items, such as age and sex; laboratory test data, such as alkaline phosphatase (ALP), calcium, and hemoglobin (Hb) concentrations, and levels of the tumor markers cancer antigen 125 (CA-125), CA-199, CA-153, alpha fetoprotein (AFP), carcinoembryonic antigen (CEA), cytokeratin fraction 21-1 (CYFRA21-1), total prostate-specific antigen (TPSA), and neuron-specific enolase (NSE); auxiliary examination data, such as histological type; and relevant diagnosis and treatment history, such as treatment methods. Then we screened out the tumor markers with significant changes in patients with liver cancer, and explored the ocular metastasis of liver cancer in patients with hypertension according to the fluctuation of their concentration.

### Statistical Analysis

SPSS 25.0 (SPSS, IBM, USA), MedCalc 19.0.5 (MedCalc Ostend, Belgium), and Excel 2019 (Microsoft Crop, Redmond, WA, USA) software were applied for statistical analyses. The Student's *t*-test and chi square test were used to evaluate whether differences in clinical features between the OM and the NM groups were statistically significant. To identify risk factors for OM of lung cancer, binary logistic regression analysis was conducted. Receiver operating characteristic (ROC) curves were plotted to estimate the accuracy and predictive value of diagnostic indicators. *P* < 0.05 was considered statistically significant.

## Results

### Demographic and Clinical Characteristics

**Figure 1:**
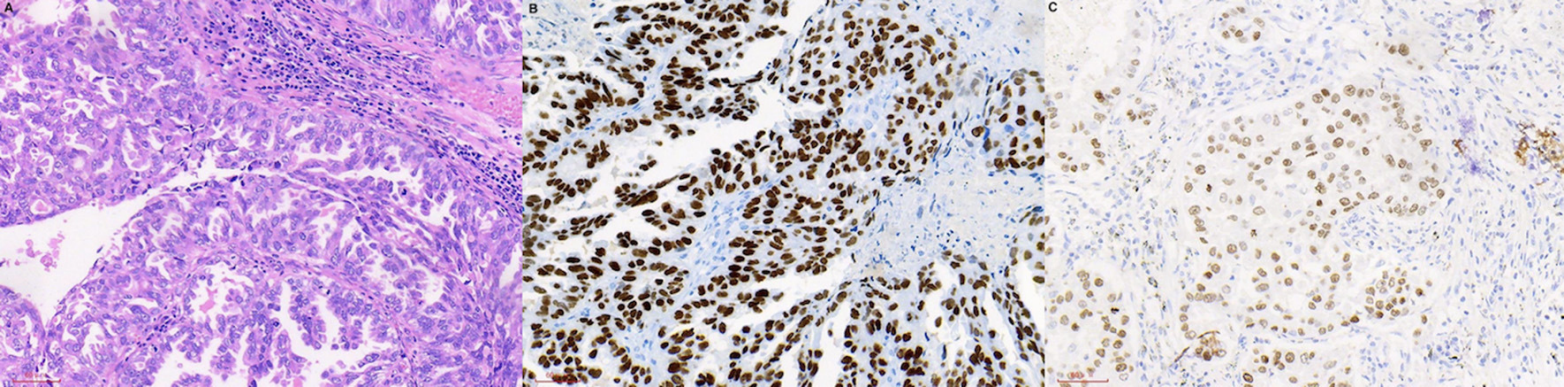
HE and IHC staining of lung cancer tissues. **(A)** HE staining. **(B)** TTF-1 (IHC staining). **(C)** CK-7 (IHC staining). HE, hematoxylin-eosin; IHC, immunohistochemistry; TTF, transcription factor; CK, creatine kinase.

**Figure 2:**
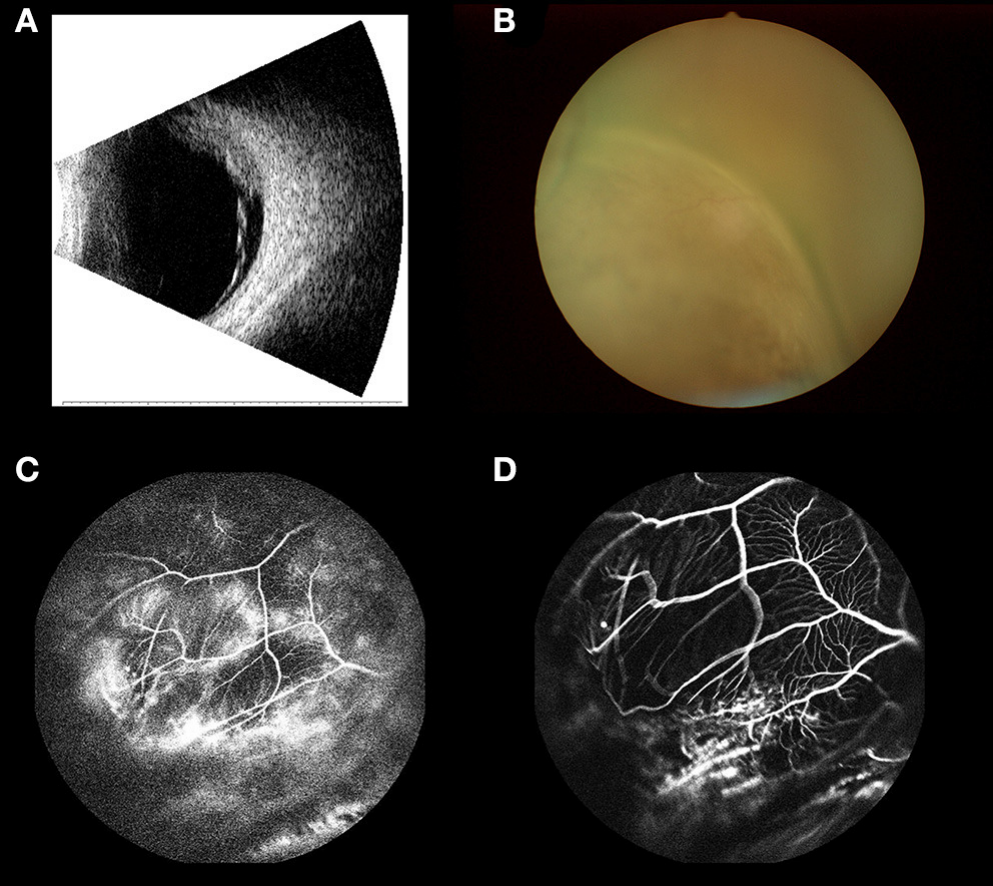
Ophthalmic imaging (ocular B-ultrasound, fundus photography, ICGA FFA) in patients with lung cancer ocular metastasis. **(A)** Ocular B-ultrasound. **(B)** Fundus photography. **(C)** ICGA. **(D)** FFA. ICGA, indocyanine green angiography; FFA, fundus fluorescein angiography.

**Figure 3:**
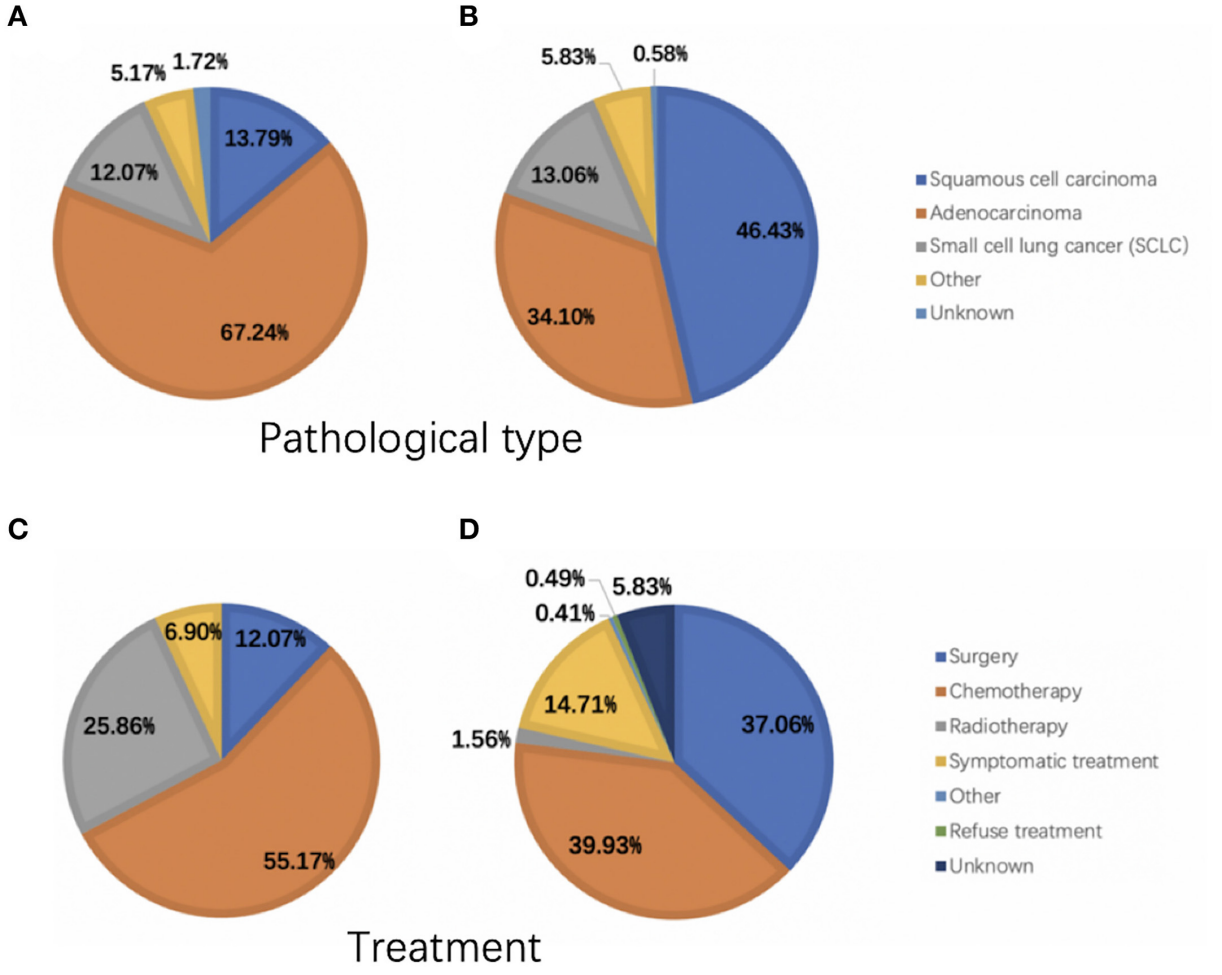
Pathological tumor types and treatment of patients in the OM and NM groups. **(A)** The pathological type of OM group. **(B)** The pathological type of NM group. **(C)** The treatment of OM group. **(D)** The treatment of NM group. OM, ocular metastasis; NM, no metastasis.

A total of 1,275 subjects participated in our trial, comprising 58 and 1,217 in the OM and NM groups, respectively. Figure 1 showed the HE and IHC staining of lung cancer tissues. Figure 2 showed the results of ophthalmic imaging (ICGA, FFA, fundus photography, ocular B-ultrasound) in patients with lung cancer ocular metastasis. The mean ages of the two groups were 59.67 ± 9.11 and 60.57 ± 10.39 years, respectively, which was not significantly different (Student's *t*-test). Further, the sex distributions of the two groups did not differ significantly. In contrast, tumor histological classification and treatment methods did differ significantly between the OM and NM groups. Adenocarcinoma was the main tumor type in the OM group (67.24%), while squamous cell carcinoma was the most common in the NM group (46.43%). Further patients in the OM group were most commonly treated with chemotherapy (55.17%), while chemotherapy (39.93%) and surgery (37.06%) were dominant therapies in the NM group (Figure 3). Detailed data on clinical characteristics are presented in Table 1.

**Table 1:** Clinical characteristics of patients with ocular metastasis of lung cancer.

**Patient characteristic**	**OM group (%)** **(*n* = 58)**	**NM group (%)** **(*n* = 1217)**	***P*-value^c^**
**Sex^a^**			
Male	43 (74.14)	913 (75.03)	0.879
Female	15 (25.86)	304 (24.97)	
Mean age (years)^b^	59.67 ± 9.11	60.57 ± 10.39	0.633
**Pathological type^a^**			
Squamous cell carcinoma	8 (13.79)	565 (46.43)	<0.001
Adenocarcinoma	39 (67.24)	415 (34.10)	
Small cell lung cancer (SCLC)	7 (12.07)	159 (13.06)	
**Other**	3 (5.17)	71 (5.83)	
Unknown	1 (1.72)	7 (0.58)	
**Treatment^a^**			
Surgery	7 (12.07)	451 (37.06)	<0.001
Chemotherapy	32 (55.17)	486 (39.93)	
Radiotherapy	15 (25.86)	19 (1.56)	
Symptomatic treatment	4 (6.90)	179 (14.71)	
Other	0 (0)	5 (0.41)	
Refused treatment	0 (0)	6 (0.49)	
Unknown	0 (0)	71 (5.83)	

a *Chi-square test*.

b *Student's t-test*.

c *Comparison between OM and NM groups. P < 0.05 was considered statistically significant. OM, ocular metastasis; NM, no metastasis*.

### Differences in Serological Indicators Between OM and NM Groups as Risk Factors for OM

Blood calcium concentration and NSE did not differ between the OM and NM groups; however, compared with the NM group, alkaline phosphatase, hemoglobin, CA-125, CA-199, CA-153, AFP, CEA, CYFRA21-1, and TPSA were significantly higher in the OM group (*p* < 0.05); detailed results are presented in Table 2. Binary logistic regression analysis demonstrated that CA-199, CA-153, AFP, CEA, and CYFRA21-1 were independent risk factors for lung cancer metastasis (Table 3).

**Table 2:** Serological indicators of ocular metastasis of lung cancer.

**Serological indicator**	**OM group**	**NM group**	***t***	***P*-value**
ALP (U/L)	108.45 ± 56.36	85.27 ± 46.20	3.083	0.003
Hb (g/L)	115.07 ± 19.74	121.10 ± 18.69	−2.395	0.017
Calcium (mmol/L)	2.26 ± 0.20	2.34 ± 2.27	−2.47	0.805
**Tumor markers**				
CA-125 (U/ml)	296.09 ± 451.18	44.25 ± 128.54	4.243	<0.001
CA-199 (U/ml)	126.52 ± 290.02	18.50 ± 37.29	2.835	0.006
CA-153 (U/ml)	71.88 ± 108.77	15.96 ± 21.40	3.912	<0.001
AFP (ng/ml)	2.89 ± 1.94	1.06 ± 1.24	7.117	<0.001
CEA (ng/ml)	218.35 ± 509.69	22.20 ± 133.47	2.926	0.005
CYFRA21-1 (ng/ml)	35.29 ± 36.70	5.81 ± 16.76	6.086	<0.001
TPSA (ng/L)	3.70 ± 2.43	1.44 ± 2.41	6.914	<0.001
NSE (U/mL)	115.07 ± 19.74	121.10 ± 18.69	1.902	0.057

*The significance of differences was evaluated using the Student's t-test, where P < 0.05 indicated statistical significance*.

*OM, ocular metastasis; NM, no metastasis; ALP, Alkaline phosphatase; Hb, hemoglobin; CA, cancer antigen; AFP, alpha fetoprotein; CEA, carcinoembryonic antigen; CYFRA, cytokeratin fraction; TPSA, total prostate-specific antigen; NSE, neuron-specific enolase*.

**Table 3:** Results of binary logistic regression to identify risk factors for ocular metastasis of lung cancer.

**Factor**	**B**	**Exp(B)**	**OR (95% CI)**	***P***
ALP	0.003	1.003	0.998–1.008	0.277
Hb	−0.010	0.990	0.972–1.008	0.274
CA-125	0.001	1.001	1.000–1.002	0.139
CA-199	0.004	1.004	1.001–1.008	0.022
CA-153	0.011	1.011	1.005–1.018	<0.001
AFP	0.675	1.964	1.555–2.481	<0.001
CEA	0.001	1.001	1.001–1.002	0.001
CYFRA21-1	0.018	1.018	1.010–1.027	<0.001
TPSA	0.128	1.137	0.972–1.330	0.108

*P < 0.05 was considered statistically significant*.

*B, coefficient of regression; OR, odds ratio; CI, confidence interval; ALP, Alkaline phosphatase; Hb, hemoglobin; CA, cancer antigen; AFP, alpha fetoprotein; CEA, carcinoembryonic antigen; CYFRA, cytokeratin fraction; TPSA, total prostate-specific antigen; NSE, neuron-specific enolase*.

### Sensitivity, Specificity, AUC, and Cut-Off Values of Serological Markers for Diagnosis of Ocular Metastasis of Lung Cancer

**Figure 4:**
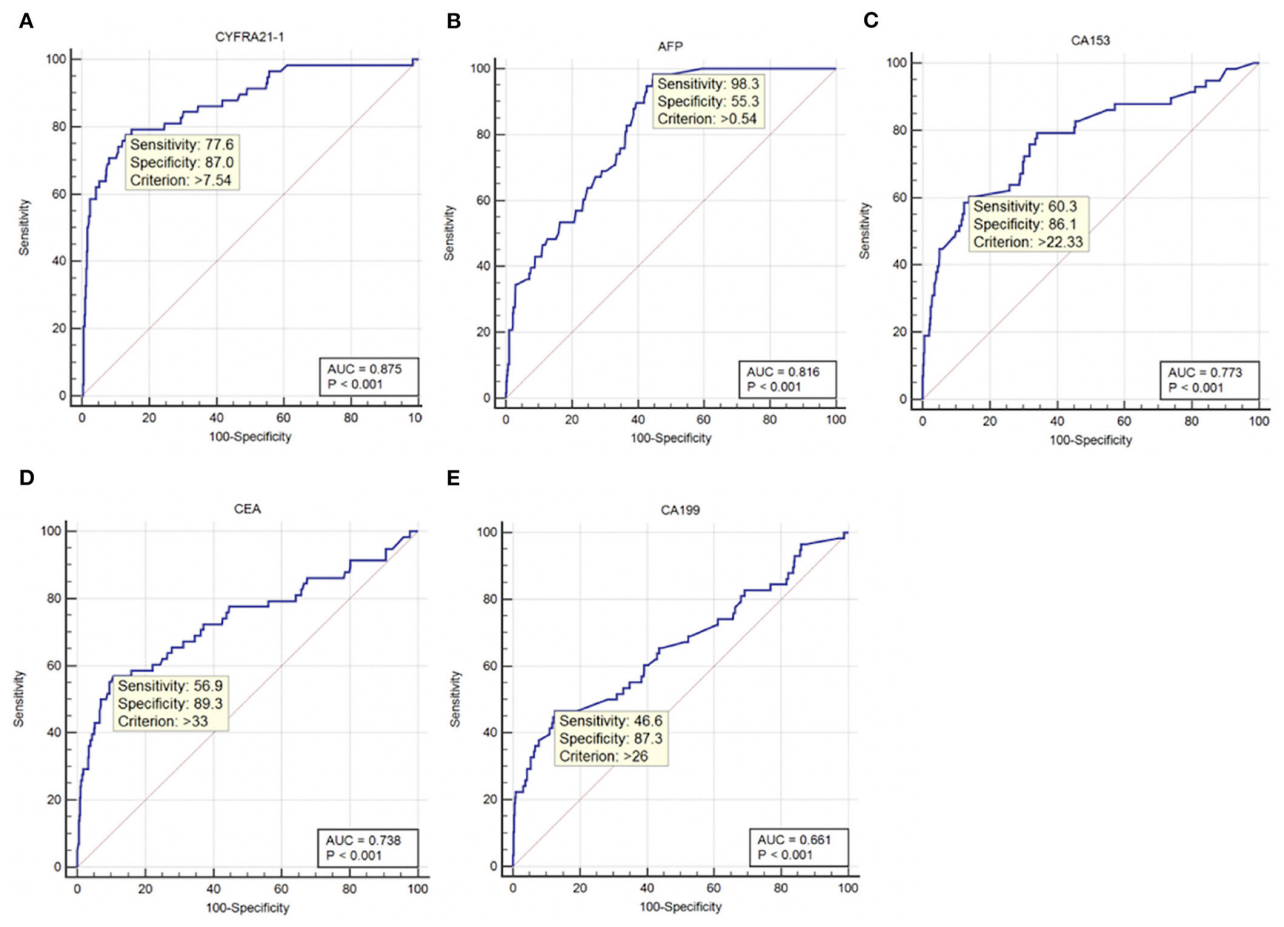
ROC curves for individual tumor markers as independent risk factors. **(A)** CYFRA21-1. **(B)** AFP. **(C)** CA-153. **(D)** CEA. **(E)** CA-199. ROC, receiver operating characteristic; AUC, area under the ROC curve; CYFRA, cytokeratin fraction; AFP, alpha fetoprotein; CA, cancer antigen; CEA, carcinoembryonic antigen.

**Figure 5:**
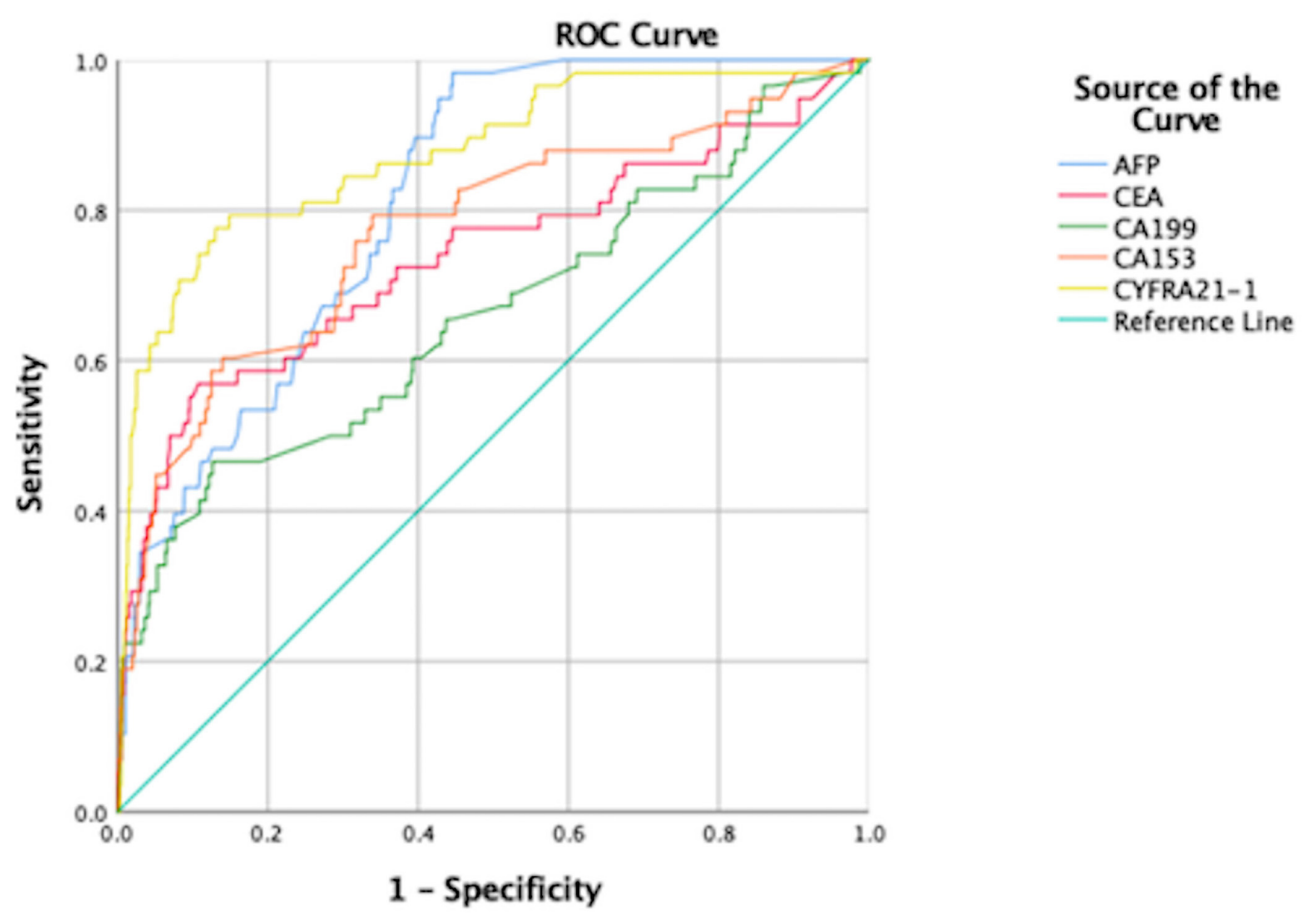
Comparison of ROC curves for tumor markers as independent risk factors. AUC, area under the ROC curve; ROC, receiver operating characteristic curve; AFP, alpha fetoprotein; CEA, carcinoembryonic antigen; CA, cancer antigen; CYFRA, cytokeratin fraction.

ROC curve analyses were performed to determine the sensitivity, specificity, area under the ROC curve (AUC) and cut-off values for CA-199, CA-153, AFP, CEA, and CYFRA21-1 (presented in Table 4, ranked in order of AUC value, from largest to smallest). The results show that AFP was clearly more sensitive than the other indicators, with a value of 98.3%. In terms of specificity, CEA had the highest value (89.3%), followed by CA-199 (87.3%), CYFRA21-1 (87.0%), and CA-153 (86.1%). The highest AUC value was for CYFRA21-1 (0.875), followed by AFP (0.816). Respective and comparative ROC curves for these tumor markers are presented in Figures 4, 5.

**Table 4:** Sensitivity, specificity, AUC, and cut-off values for tumor markers as diagnostic indicators.

**Factor**	**Sensitivity (%)**	**Specificity (%)**	**AUC**	**Cut-off value**	***P***
CYFRA21-1	77.6	87	0.875	7.54 ng/ml	<0.001
AFP	98.3	55.3	0.816	0.54 ng/ml	<0.001
CA-153	60.3	86.1	0.773	22.33 U/ml	<0.001
CEA	56.9	89.3	0.738	33 ng/ml	<0.001
CA-199	46.6	87.3	0.661	26 U/ml	<0.001

*Sensitivity and specificity were obtained at the cut-off value. P < 0.05 was considered statistically significant*.

*AUC, area under the curve; CYFRA, cytokeratin fraction; AFP, alpha fetoprotein; CA, cancer antigen; CEA, carcinoembryonic antigen*.

## Discussion

Lung cancer, a common disease in China, is ranked the third leading cause of years of life lost, after age-standardized stroke and ischemic heart disease (18). Tobacco smoking is a major risk factor for lung cancer, with other contributory factors including genetic susceptibility, occupational exposures, poor diet, and air pollution (19). According to histological type, lung cancer can be classified as NSCLC or SCLC, and NSCLC further subdivided into adenocarcinoma, squamous cell carcinoma, large cell carcinoma, and non-specific tumor type (5, 20). Su et al. (21) previously found that adenocarcinoma is more likely to lead to OM, which is consistent with our findings.

Metastasis is primarily responsible for lung cancer-related morbidity and mortality (22). Unfortunately, due to lack of accurate early detection, patients are usually diagnosed at an advanced disease stage (15). Bone and brain are among the most common metastasis sites (9); however, the eye is a rare site for disseminated lung cancer, as it is isolated from the lymphatic system. The malignancy invades the eye through hematogenous spread, and the choroid, because of its abundant blood flow, is more likely to be affected during advanced stage disease, while uveal metastases can occur at an earlier stage (23). The symptoms of ocular metastases include decreased vision, visual field defects, pain, muscae volitantes, and elevated intraocular pressure (16); however, most patients with ocular metastases have no obvious symptoms (21), which delays clinical diagnosis and treatment, resulting in poor prognosis.

Most patients in the OM group received chemotherapy (55.17%), while those in the NM group had chemotherapy (39.93%) and surgery (37.06%). Surgery is the first-choice for treatment of lung cancer (24), with survival rates being 80% better than those of other therapies (25); however, patients with advanced disease are usually not indicated for surgery, and are generally administered chemotherapy, targeted therapies, and/or somatostatin analog therapy as drug-based approaches, or peptide receptor radionuclide therapy (24). In particular, small cell lung cancer is not generally indicated for surgery (26), with radiation therapy or chemotherapy as the main treatment methods (27).

Although conventional tumor diagnosis methods are highly accurate, they require invasive examination and are expensive, which is not conducive to early screening for tumors. Tumor marker evaluation is non-invasive, safe, inexpensive, and simple; hence, it is more suitable for tumor screening (28). Commonly used tumor markers include CA-125, CA-153, CA-199, AFP, CEA, CYFRA21-1, TPSA, and NES, among others. CA-125, CA-199, and CA-153 are markers for ovarian, pancreatic or gastrointestinal, and breast cancer, respectively (29–32), while AFP is a tumor marker characteristic of liver cancer (33). In addition, AFP is increased in patients with liver and brain metastasis of lung cancer (34, 35). CEA is an important marker of colon cancer (36) and is also reported to be a highly sensitive marker for lung cancer assessment (37), particularly adenocarcinoma and squamous cell carcinoma (38). TPSA can be applied for assessment of prostate cancer (39), while CYFRA21-1 is a new biomarker with value for diagnosis of NSCLC (40), particularly squamous cell carcinoma (41). In addition, thymic carcinoma (42), colorectal cancer (43), and bladder cancer (44) are associated with increased CYFRA21-1 levels. NSE can be a sensitive indicator for SCLC (39). Further, these markers can be used in combination for lung cancer diagnosis (45, 46), and researchers have also confirmed that they can be used in combination or alone to screen for lung cancer metastases. Research reports of tumor markers for metastatic lung cancer sites published in recent years are presented in Table 5. Notably, Lin et al. studied patients with lung cancer with metastases other than the eyes as a control group, and concluded that CA-153 and CYFRA21-1 were risk factors for ocular metastasis of lung cancer. Our subjects are all in hypertension population, which is more consistent with the incidence of lung cancer. We included lung cancer patients without metastatic lesions as controls in contrast. Furthermore, serum ALP, serum calcium, and Hb concentrations were used to evaluate liver, bone, and nutritional status. We attempted to combine the above markers to assess tumor metastasis and the general condition of subjects. Our results show that CYFRA21-1, AFP, CA-153, CEA, and CA-199 levels are associated with OM of lung cancer, and can be used as independent risk factors. Their respective ROC curves were used to evaluate their accuracy as diagnostic indicators. The sensitivity of AFP was much higher than that of other markers, at 98.3%. In terms of specificity, CEA was highest (89.3%), but CA-199 (87.3%), CYFRA21-1 (87.0%), and CA-153 (86.1%) were similarly specific. The two indicators with the highest AUC values were CYFRA21-1 and AFP, at 0.875 and 0.816, respectively. Based on these data, AFP is a suitable marker for screening for OM of lung cancer, with risk of OM increased in patients with serum concentrations of AFP ≥ 0.54 ng/ml. CYFRA21-1 is the most accurate indicator of lung cancer OM, which should be evaluated by imaging in patients with serum levels >7.54 ng/ml.

**Table 5:** Tumor markers for metastatic lung cancer sites.

**Author**	**Year**	**Histopathological Type**	**Metastatic sites**	**Risk factor**
Pollán et al. (47)	2003	NSCLC	NS	p53, c-erbB-2, CEA, CA125
Arrieta et al. (48)	2009	NSCLC	Brain	CEA
Cabrera-Alarcon et al. (49)	2011	NSCLC	NS	CYFRA 21-1
Chen et al. (50)	2015	NS	Lymph node	CYFRA 21-1,CEA
Chen et al. (51)	2015	NSCLC	Brain	NSE
Zhou et al. (52)	2017	NS	Bone	CA-125, ALP
Morita et al. (53)	2019	SCLC	Intertrabecular Vertebral	CEA
Lin et al. (54)	2020	NS	Intraocular	CA153, CYFRA 21-1

*NS, not specific; NSCLC, non-small cell lung cancer; SCLC, small cell lung cancer; CEA, carcinoembryonic antigen; NSE, serum neuron-specific enolase; ALP, alkaline phosphate; CA, cancer antigen*.

Our research has some limitations. First, as some patients with OM also had metastases at other sites, it is difficult to rule out confounding factors. Second, the number of subjects in our trial is relatively small, particularly in the OM group. Furthermore, while we statistically evaluated differences in histological types and treatments between the OM and NM groups, we did not specifically study their effects on lung cancer metastasis. At the same time, the pathological stages of samples have not been strictly controlled, which needs further study. Finally, all patients were diagnosed and treated in the same hospital, hence there may be selection bias. Therefore, a multi-center, large-sample, prospective study is required to test our conclusions.

## Conclusion

The eye is a rare site for lung cancer metastasis. Patients in hypertension population with adenocarcinomas, primarily treated with chemotherapy are more likely to develop OM. Further, CYFRA21-1, AFP, CA-153, CEA, and CA-199 are independent risk factors for OM, among which, CYFRA21-1 is the most promising diagnostic indicator.

## Data Availability Statement

The data that support the findings of this study are available from the corresponding author upon reasonable request.

## Ethics Statement

The studies involving human participants were reviewed and approved by the First Affiliated Hospital of Nanchang University. The patients/participants provided their written informed consent to participate in this study.

## Author Contributions

JT and Q-MG was responsible for conceiving and designing the work, acquiring the data, and writing the manuscript. RH played an important role in interpreting the results and perform the analysis with constructive discussions. H-YS helped acquire data and gave some advice. TS helped make the figures. J-LW contributed to helping make the tables. Y-CP, R-BL, and L-JZ revised the manuscript. YS and YY helped design the work and approved the final version. All authors contributed to the article and approved the submitted version.

## Funding

This work was supported by the National Natural Science Foundation of China (Nos: 81660158, 81460092, and 81400372); Natural Science Key Project of Jiangxi Province (No: 20161ACB21017); Health Development Planning Commission Science Foundation of Jiangxi Province (No: 20175116); National Natural Science Foundation (No: 82160195); Central Government Guides Local Science and Technology Development Foundation (No: 20211ZDG02003); Key Research Foundation of Jiangxi Province (No: 20203BBG73059, 20181BBG70004); Excellent Talents Development Project of jiangxi Province (20192BCBL23020); Natural Science Foundation of jiangxi Province (20181BAB205034); Health Development Planning Commission Science Foundation of Jiangxi Province (No: 20201032).

## Conflict of Interest

The authors declare that the research was conducted in the absence of any commercial or financial relationships that could be construed as a potential conflict of interest.

## Publisher's Note

All claims expressed in this article are solely those of the authors and do not necessarily represent those of their affiliated organizations, or those of the publisher, the editors and the reviewers. Any product that may be evaluated in this article, or claim that may be made by its manufacturer, is not guaranteed or endorsed by the publisher.
